# Management and outcomes of breast cancer patients with radiotherapy interruption

**DOI:** 10.3389/fonc.2024.1337194

**Published:** 2024-07-23

**Authors:** Fangrui Zhao, Dashuai Yang, Yanfang Lan, Xiangpan Li

**Affiliations:** ^1^ Department of Oncology, Renmin Hospital of Wuhan University, Wuhan, Hubei, China; ^2^ Department of Hepatobiliary Surgery, Renmin Hospital of Wuhan University, Wuhan, Hubei, China

**Keywords:** breast cancer, radiotherapy, interruption, COVID-19, prognosis

## Abstract

**Background:**

Many cancer patients have not received timely treatment or even had treatment interruptions due to the COVID-19 pandemic. The objective of this investigation was to evaluate whether the prognosis of patients with breast cancer after surgery was affected by any interruptions in radiotherapy.

**Methods:**

The healthcare documents for breast cancer patients experiencing radiotherapy interruption after surgery, including treatment-related characteristics, and time of interruption, type of disease progression, and survival status, were collected between January and April 2020 during the Wuhan blockade.

**Results:**

The final number of patients included was 148, and neither the Kaplan-Meier (KM) survival curve nor the cross-tabulation analysis found statistical significance. Cox regression analysis also did not identify risk factors associated with PFS.

**Conclusions:**

The prognosis of patients with postoperative breast cancer may not be significantly impacted by the interruption of radiotherapy, given its integration with additional treatments like targeted and endocrine therapies.

## Background

Breast cancer has become more prevalent than lung cancer, ranking as the fifth most common type of cancer and the fifth leading cause of cancer-related fatalities globally (1). According to statistics, approximately 2.3 million people will be diagnosed with breast cancer in 2020, and 685,000 people will die from breast cancer (2). The incidence of breast cancer is projected to reach 4.4 million cases by 2070 (3). Breast cancer accounts for approximately 24.5% of all cancer cases in women, making it the leading cause of global cancer-related deaths, representing approximately 15.5%. It ranks first in most countries in the world in terms of incidence and mortality in 2020 (1).

The classification of breast cancer is primarily based on the presence or absence of estrogen receptor (ER), progesterone receptor (PR), and human epidermal growth factor receptor 2 (HER2), reflecting its diverse nature. These distinct subtypes exhibit variations in etiology and prognostic outcomes (4). The most prevalent subtype of breast cancer is characterized by being estrogen receptor (ER) and/or progesterone receptor (PR) positive while lacking human epidermal growth factor receptor 2 (HER2), accounting for approximately 73% of all breast cancers (5).

The selection of the treatment protocol should rely on the patient’s molecular profile. The treatment strategies vary depending on the stage of breast cancer. Surgical management of ductual carcinoma *in situ* includes breast conserving surgery (lumpectomy), often followed by radiotherapy, or mastectomy, after which radiotherapy is not indicated, aside from rare cases of positive margin when re-resection cannot be done. When ER is positive, endocrine therapy may also be an option. Depending on its biological profile, invasive ductal carcinoma may require endocrine therapy, targeted agents, immunotherapy, and/or chemotherapy, in the neoadjuvant or adjuvant setting (6). Triple-negative breast cancer almost always is an indication for chemotherapy, as it is typically the most aggressive subtype of breast cancer.

Radiotherapy has become an essential part of breast cancer treatment. Postoperative radiation therapy has the potential to eliminate any remaining tumor cells, leading to enhanced control rates of both local (primary site) and/or regional (axillary nodal basin) diseases (7, 8) and minimizing the need for extensive surgical procedures (9–11). It has been shown that the 10-year local recurrence rate and distant metastasis rate were reduced by approximately 16% and the risk of death by approximately 4% in female patients with negative lymph nodes after breast-conserving surgery who received radiotherapy (8). Patients who have experienced a recurrence of breast cancer in the same area can also achieve favorable outcomes through re-administration of radiotherapy (12–14). Studies have demonstrated that reirradiation can lead to high rates of local control, ranging from 43% to 96%, and varying complete response rates between 41% and 71% (14).

Coronavirus disease 2019 (COVID-19) emerged in December 2019, and the World Health Organization declared that it reached pandemic status on 11 March 2020 (15, 16). Due to the highly infectious nature of the virus, isolation and reduction in crowd gathering became the mainstream response until new treatments and vaccines were available (17). The outbreak of COVID-19 not only led to the suspension of many social events and closure of public facilities but also had a dramatic impact on healthcare services (18). Many cancer patients progressed or even died due to delayed diagnosis, untimely treatment, or even interruption (19, 20). Radiotherapy is a crucial component in the management of breast cancer, and it remains to be studied whether interruption of radiotherapy due to an epidemic outbreak can cause disease progression.

## Methods

### Patients

Clinical data of all breast cancer patients who received radiotherapy (interrupted or uninterrupted) at 10 hospitals in Hubei province since the outbreak of the new crown epidemic (January–April 2020) were collected. The inclusion criteria were as follows: 1) patients with pathological diagnosis of invasive breast cancer and 2) patients who had undergone surgery and postoperative radiotherapy. The breast cancer patients included in this study were treated with regional nodal irradiation (RNI) in addition to breast/chest wall irradiation for node-positive patients. In addition, patients who were node-negative but clinically assessed as high risk were also treated with RNI.

Demographic, clinical, and treatment information was retrieved from each hospital’s medical records and filing system.

### Statistical analysis

The overall survival (OS) was defined as the duration from the initiation of radiotherapy following breast cancer surgery until death resulting from any cause, while progression-free survival (PFS) referred to the period from the commencement of radiotherapy after breast cancer surgery until disease progression. Patients were continuously monitored for tumor progression and survival data until their last follow-up in March 2023. Radiotherapy interruption was defined as a situation in which the treatment plan has to be temporarily suspended for some reason during the course of radiotherapy. Included patients were divided into two groups based on whether or not the interruption lasted more than 7 days, i.e., radiotherapy interrupted (RTI) and non-radiotherapy interrupted (non-RTI), using propensity score matching (PSM) to adjust for potential baseline confounders (demographic data, clinical characteristics, and treatment) to make the two groups comparable. The analysis of categorical variables involved the use of cross-tabulation and chi-square tests, while survival data estimation was conducted using Kaplan–Meier curves and log-rank tests. Cox regression analysis was employed to identify and evaluate risk factors associated with progression-free survival (PFS). Statistical significance was determined based on two-tailed p-values <0.05. All statistical analyses were performed using SPSS 24.0 (SPSS Inc., Chicago, USA).

## Results

### Radiotherapy interruption did not cause survival difference

We collected data related to radiotherapy for breast cancer patients in 10 hospitals in Hubei province, and all patients were women. The total number of patients included through PSM was 148, with 74 patients in each of the RTI and non-RTI groups. The details of the included patients are shown in **Table 1**. The reason for the interruption of radiotherapy is mainly due to the COVID-19 outbreak, which has caused regional lockdown and prevented patients from travelling normally. The average age of individuals in the RTI group was recorded as 48 years, while those in the non-RTI group had an average age of 50 years. The proportion of patients ≤50 years old was the same in both groups. In the RTI group, 18% of patients were clinically staged as stage I, 43% as stage II, and 38% as stage III. In the non-RTI group, the proportions of patients clinically staged as stage I, stage II, and stage III were 12%, 42%, and 45%, respectively. Only a small proportion of included patients received neoadjuvant chemotherapy (RTI, 27%; non-RTI, 14%), targeted therapy (RTI, 23%; non-RTI, 27%), and endocrine therapy (RTI, 36%; non-RTI, 34%). However, the use of neoadjuvant chemotherapy was almost twice as high in the RTI (vs. non-RTI) group.

**Table 1:** Baseline characteristics of postoperative breast cancer patients.

	Group			
	Non-RTI		RTI	
**Median age (years old)**	48		50	
Age				
≤50	43	58%	43	58%
>50	31	42%	31	42%
TNM				
I	13	18%	9	12%
II	32	43%	31	42%
III	28	38%	33	45%
IV	1	1%	1	1%
Neoadjuvant chemotherapy				
No	54	73%	64	86%
Yes	20	27%	10	14%
Adjuvant chemotherapy				
No	30	41%	20	27%
Yes	44	59%	54	73%
Targeted therapy				
No	57	77%	54	73%
Yes	17	23%	20	27%
Endocrinotherapy				
No	47	64%	49	66%
Yes	27	36%	25	34%
Continued				
No	─		23	31%
Yes	─		51	69%

**Figure 1:**
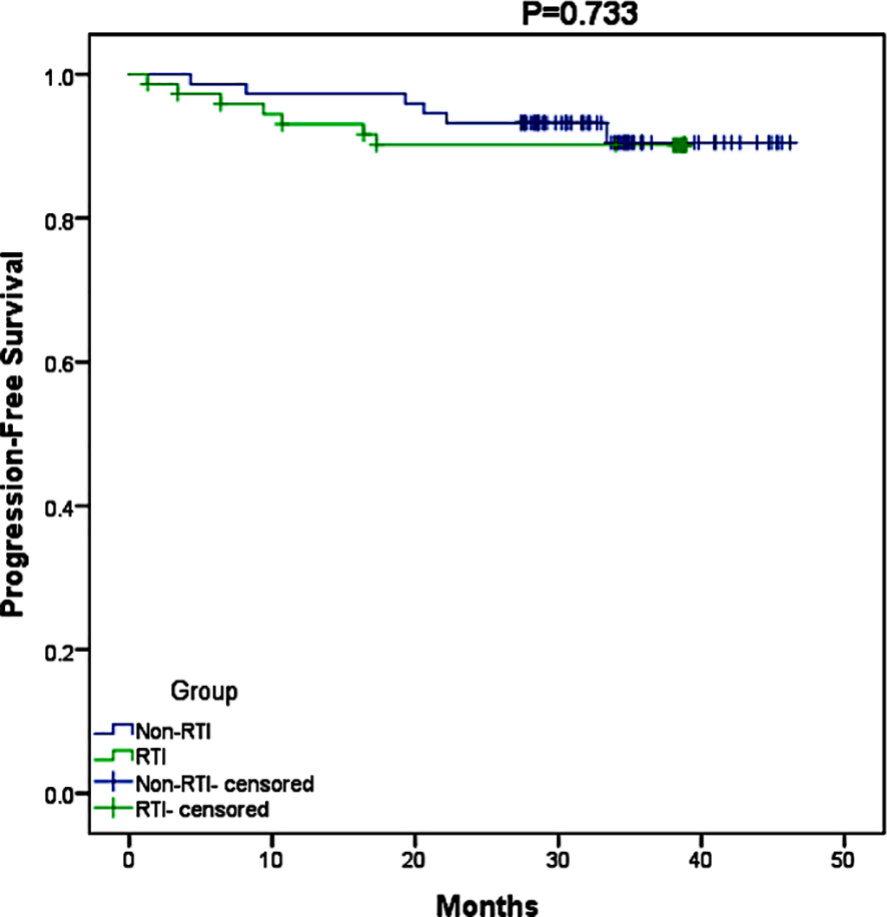
Kaplan-Meier survival curve of PFS between RTI and non-RTI.

**Figure 2:**
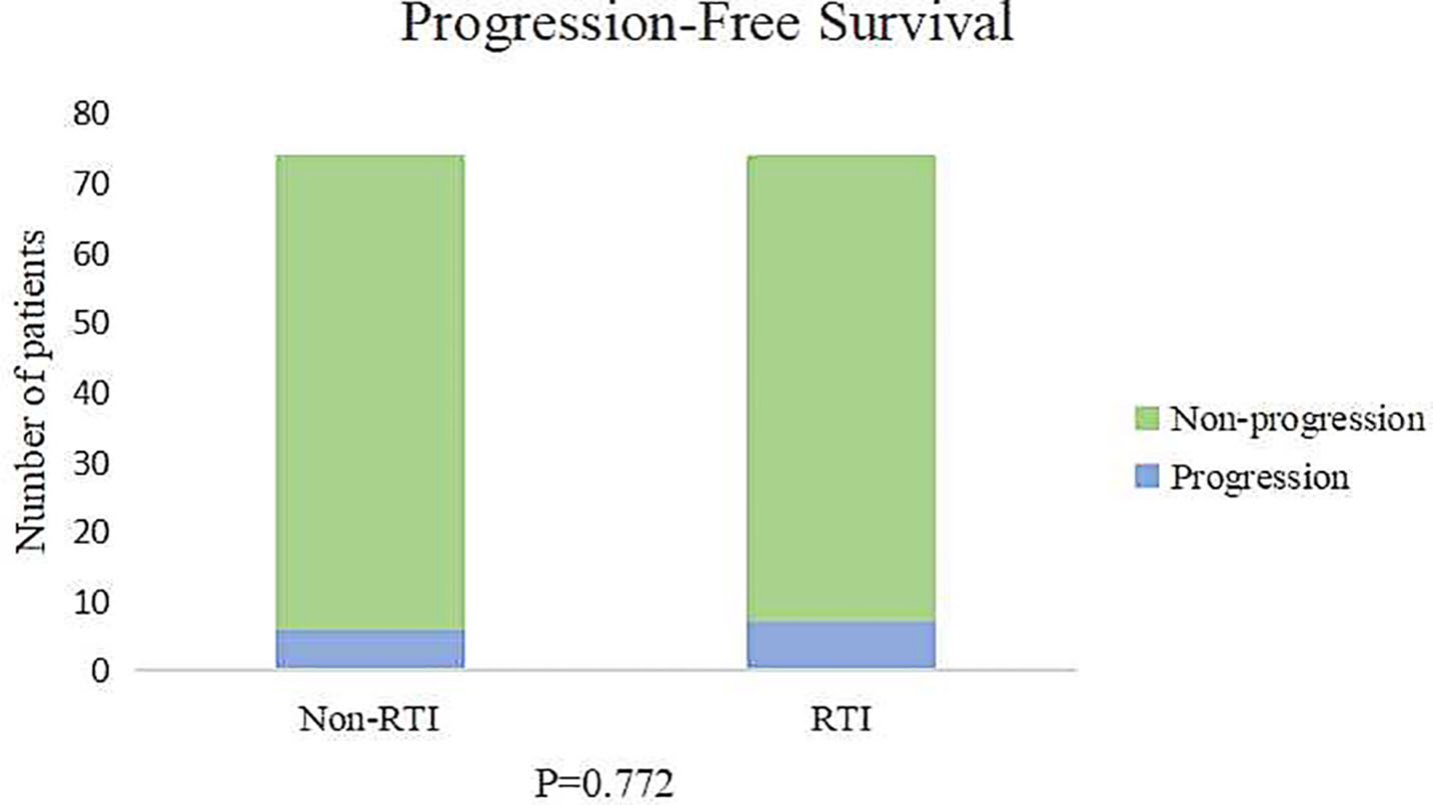
The impact of RTI on disease progression.

In the RTI group, 59% of patients received adjuvant chemotherapy, while in the non-RTI group, this percentage was higher at 73%. The median duration of follow-up (calculated from the commencement of radiotherapy until the most recent contact) was 37.3 months (ranging from 1.3 months to 46.2 months). **Figure 1** illustrates that there was no disparity in survival between the two groups due to the interruption of radiotherapy (p=0.733), and **Figure 2** further confirms that radiotherapy interruption did not affect PFS in patients after breast cancer surgery (p=0.722). In addition, the 3-year PFS rates were comparable between the two groups, with percentages of 91.9% and 90.5%, respectively.

In the non-RTI group, two patients died and four patients progressed. In contrast, in the RTI group, no patients died. Of these seven patients, only one patient did not choose to return to thehospital for further radiotherapy after the interruption of radiotherapy.

### No discernible risk factors associated with PFS were identified

**Figure 3:**
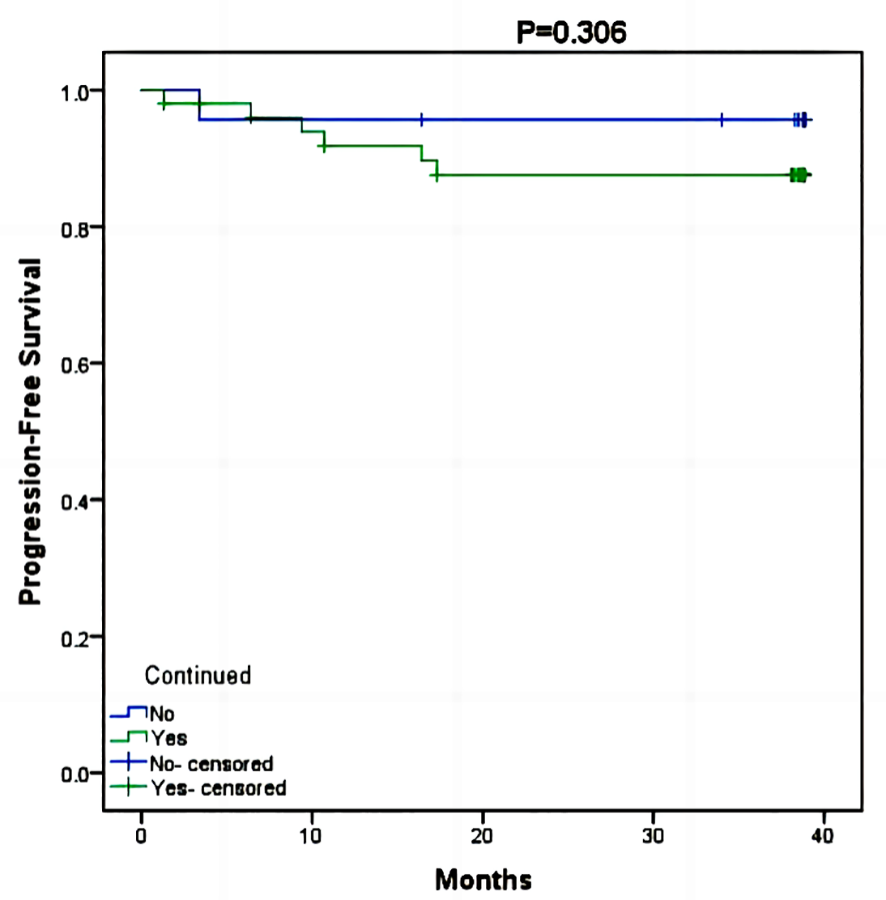
Kaplan-Meier survival curve of PFS between patients who continued radiotherapy and those who did not.

**Figure 4:**
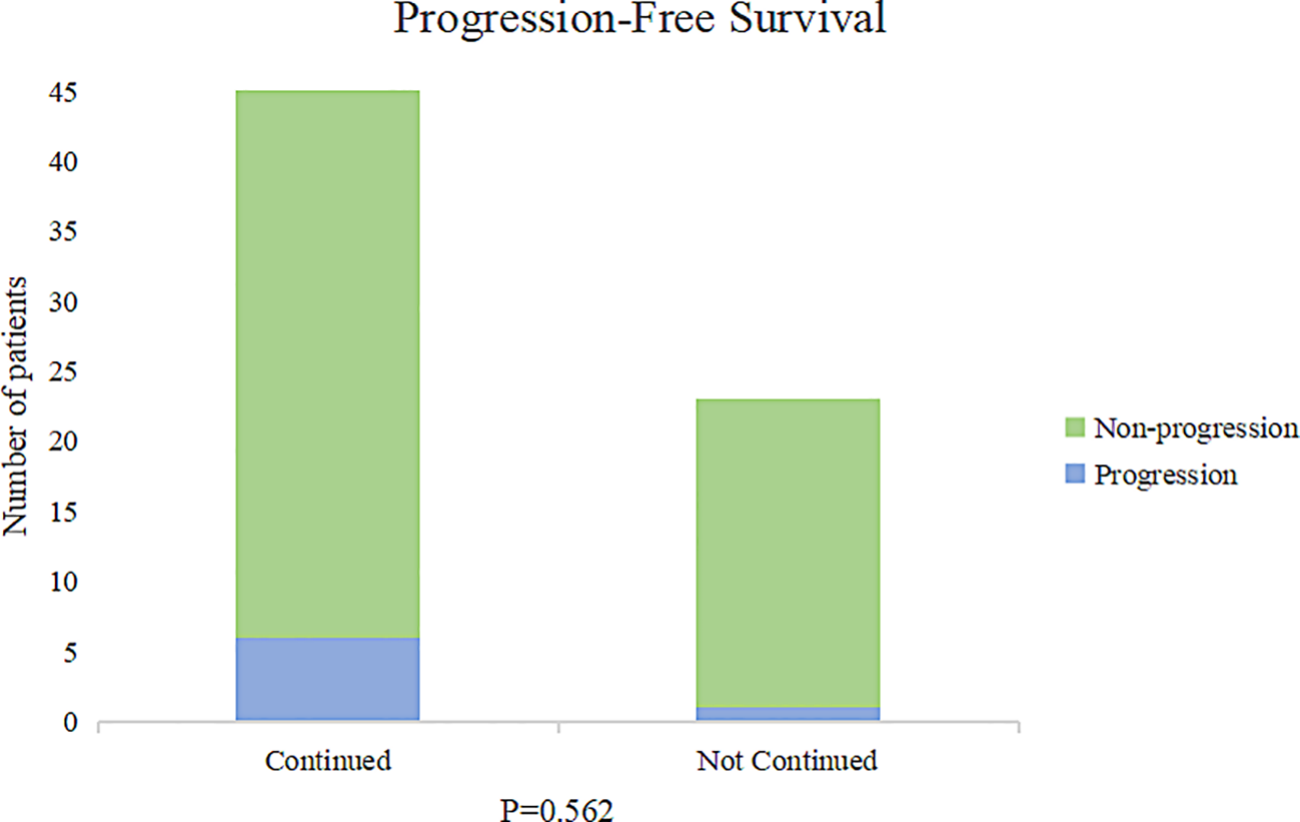
Effect of continuation of radiotherapy after RTI on disease progression.

In the RTI cohort, a majority of patients (69%) opted for hospital readmission to continue their radiotherapy treatment, with a median RTI of 78 days (ranging from 7 days to 155 days). Cox risk regression models suggested that clinical stage, neoadjuvant chemotherapy, adjuvant chemotherapy, targeted therapy, endocrine therapy, and return to continued treatment were not factors in the difference in survival (p>0.05) (**Table 2**). The KM survival curve further demonstrated that there was no difference in prognosis between patients who continued to return to radiotherapy and those who abandoned continuing radiotherapy (p=0.306) (**Figure 3**). Although there was a noticeable decrease in the 3-year PFS rate for patients who received continued radiotherapy (88.2%) compared to those who did not (95.7%), statistical analysis did not reveal any significant difference between the two groups (p=0.562) (**Figure 4**).

**Table 2:** Multivariate Cox proportional hazards regression model analysis of PFS in RTI.

Groups	p	Exp(B)	95.0% CI	
Age				
≤50				
>50	0.736	1.375	0.216	8.772
TNM	0.203			
I				
II	0.337	0.222	0.01	4.79
III	0.724	0.632	0.05	8.053
IV	0.233	8.838	0.247	316.82
Neoadjuvant chemotherapy				
No				
Yes	0.474	3.391	0.119	96.236
Adjuvant chemotherapy				
No				
Yes	0.346	4.753	0.186	121.373
Targeted therapy				
No				
Yes	0.955	0.948	0.15	5.981
Endocrinotherapy				
No				
Yes	0.428	0.406	0.044	3.772
Continued				
No				
Yes	0.461	2.368	0.239	23.487

## Discussion

Radiotherapy is typically recommended after lumpectomy for most patients with invasive breast cancer, stages I–III, and after mastectomy for women with T4 disease and/or lymph node metastases. The integration of these treatments optimizes disease management at the local level, resulting in favorable survival outcomes (21). In the last decades, radiotherapy techniques have been greatly improved (22), and the incidence of radiotherapy-induced cardiopulmonary toxicity has improved (23).

The primary mechanism of radiotherapy is to eliminate cancerous cells by causing irreversible harm to the genetic material and membranes of these cells (24). Ionizing radiation stimulates the generation of reactive oxygen species (ROS) through the breakdown of cellular components. Direct damage to DNA occurs as a result of ionizing radiation, while ROS induce DNA cross-linking, base or sugar impairment, and single- and double-strand breaks (DSBs) (25).

However, due to the COVID-19 pandemic, Wuhan went into lockdown, which also led to interruptions in breast cancer patients undergoing radiotherapy, with interruptions ranging from 6 to 155 days. In rapidly proliferating cancers, prolonging the duration of radiotherapy leads to a decrease in tumor control (26). This loss of tumor control may be related to the accelerated proliferation (repopulation) of tumor cells that survive the interruption period (27, 28). In nasopharyngeal carcinoma, radiotherapy interruptions of just more than 7 days can adversely affect patient prognosis (29, 30), and in head and neck cancers (31). However, Richard et al. noted that breast cancer patients who underwent surgery within 90 days of diagnosis and radiotherapy within 365 days did not have as great an impact on prognosis as expected (32). The impact on survival after patients with breast cancer experiencing interruption in the course of radiotherapy is, as yet, unknown.

In theory, it appears possible to evaluate the influence of radiotherapy interruption on the prognosis of breast cancer through assessing the duration for tumor cells to multiply. In practice, however, tumors initially grow at a parabolic exponential growth rate, and then, the expansion rate begins to decrease and plateau, which is related to limitations in blood supply, growth space, and nutrition, and the chaotic growth pattern of the tumor itself (32, 33). The nonlinear growth kinetic pattern of the tumor results in a tumor multiplication time that is not constant (32, 34, 35), which also causes the assessment to become unreliable. Second, the overall duration of the tumor’s existence remains indeterminable, further leading to uncertainty in the relationship between tumor multiplication time and the prognostic impact of interrupted radiotherapy (32).

The findings of this investigation indicate that the discontinuation of radiotherapy appeared to have minimal impact on individuals diagnosed with breast cancer. First, all of the patients underwent surgery, which removed the vast majority of the tumor cells, and most of the patients were treated with chemotherapy. Paclitaxel induces tumor cell aggregation in G2 and M phases to promote cell death. Although concurrent chemotherapy is not often used in the treatment of breast cancer, its synergistic effect in radiotherapy is undeniable. It has been demonstrated that the combination of a low dosage of paclitaxel effectively reduces the proliferation and survival of cancer cells when subjected to fractionated radiotherapy in lung cancer (36). Radiation exerts its effects during the G1/S and G2/M stages, while paclitaxel induces the accumulation of tumor cells in the G2 and M phases. The growth delay observed when combining paclitaxel with fractionated radiotherapy is attributed to the obstruction of various stages within the cell cycle (36, 37). Paclitaxel has been found to enhance the sensitivity of breast cancer cells to radiation by improving tumor interstitial pressure and oxygenation and promoting apoptosis (37). Furthermore, radiosensitivity in breast cancer can be increased through the use of alkylating agents like cisplatin (38) and antimetabolites such as capecitabine (39).

Patients who are HER2+ and/or ER and/or PR positive may undergo adjuvant HER2-targeted therapy and/or endocrine therapy concurrently with radiotherapy (21). The majority of patients in this study underwent endocrine therapy and/or targeted therapy, which may further compensate for the effect of radiotherapy interruption. Endocrine therapy is the cornerstone of hormone-responsive breast cancer treatment, and commonly used agents include aromatase inhibitors or tamoxifen (40). Combining RT with aromatase inhibitors or tamoxifen enhances radiosensitivity and apoptosis (41) and promotes tumor volume reduction (42, 43). Wang et al. conducted a study that discovered that the combination of endocrine therapy and RT resulted in cell redistribution within the G1 phase, leading to a significant decrease in the proportion of breast cancer cells arrested in the G2 and S phases compared to RT alone. Furthermore, an increase in cell cycle arrest was observed, along with a reduction in the regulation of DNA-PKcs, a protein involved in non-homologous repair, and RAD51, a protein involved in homologous recombination repair (44). Therefore, endocrine therapy can be used as a radiosensitizer for hormone receptor-positive breast cancer cells and can be performed in parallel with RT (45, 46).

The limitations of this study were as follows: 1) the sample size was limited, and only postoperative breast cancer patients were included in the study; 2) the duration of follow-up was insufficient, potentially leading to undetected recurrence and metastasis of tumors, necessitating further observation; 3) differences in survival would take many more years to detect, but this is most likely limited by the sample size of this study and the length of follow-up.

## Conclusions

The period of the COVID-19 pandemic and the implementation of a lockdown in Wuhan from 23 January to 8 April 2020 provided an exceptional opportunity to assess the impact of contemporary interruptions in radiotherapy. This study offered the most comprehensive follow-up to date for evaluating outcomes associated with interruption of radiotherapy. The results of the study analysis suggest that radiotherapy interruption did not have an impact on the short-term outcomes of postoperative breast cancer patients, which could be due in part to the combination with other treatment modalities. However, what a great risk reduction tool radiation is in the adjuvant (post-op) setting in breast cancer. Therefore, the importance of timely action should not be undermined when RTI happens.

## Data availability statement

The raw data supporting the conclusions of this article will be made available by the authors, without undue reservation.

## Ethics statement

The studies involving humans were approved by The Ethics Committee of Renmin Hospital of Wuhan University. The studies were conducted in accordance with the local legislation and institutional requirements. Written informed consent for participation was not required from the participants or the participants’ legal guardians/next of kin because this was a retrospective study, so informed consent was not required.

## Author contributions

FZ: Software, Writing – original draft. DY: Formal analysis, Methodology, Writing – original draft. YL: Conceptualization, Data curation, Writing – review & editing. XL: Writing – review & editing.

## References

[1] Lei S Zheng R Zhang S Wang S Chen R Sun K. Global patterns of breast cancer incidence and mortality: A population-based cancer registry data analysis from 2000 to 2020. Cancer Commun (Lond). (2021) 41:1183–94. doi: 10.1002/cac2.12207 PMC8626596 34399040

[2] Sung H Ferlay J Siegel RL Laversanne M Soerjomataram I Jemal A. Global cancer statistics 2020: GLOBOCAN estimates of incidence and mortality worldwide for 36 cancers in 185 countries. CA Cancer J Clin. (2021) 71:209–49. doi: 10.3322/caac.21660 33538338

[3] Soerjomataram I Bray F. Planning for tomorrow: global cancer incidence and the role of prevention 2020-2070. Nat Rev Clin Oncol. (2021) 18:663–72. doi: 10.1038/s41571-021-00514-z 34079102

[4] McCarthy AM Friebel-Klingner T Ehsan S He W Welch M Chen J. Relationship of established risk factors with breast cancer subtypes. Cancer Med. (2021) 10:6456–67. doi: 10.1002/cam4.4158 PMC8446564 34464510

[5] Howlader N Altekruse SF Li CI Chen VW Clarke CA Ries LA. US incidence of breast cancer subtypes defined by joint hormone receptor and HER2 status. J Natl Cancer Inst. (2014) 106(5):dju055. doi: 10.1093/jnci/dju055 PMC4580552 24777111

[6] Trayes KP Cokenakes SEH. Breast cancer treatment. Am Fam Physician. (2021) 104:171–8. 34383430

[7] Ebctcg McGale P Taylor C Correa C Cutter D Duane F. Effect of radiotherapy after mastectomy and axillary surgery on 10-year recurrence and 20-year breast cancer mortality: meta-analysis of individual patient data for 8135 women in 22 randomised trials. Lancet. (2014) 383:2127–35. doi: 10.1016/S0140-6736(14)60488-8 PMC5015598 24656685

[8] G. Early Breast Cancer Trialists' Collaborative Darby S McGale P Correa C Taylor C Arriagada R. Effect of radiotherapy after breast-conserving surgery on 10-year recurrence and 15-year breast cancer death: meta-analysis of individual patient data for 10,801 women in 17 randomised trials. Lancet. (2011) 378:1707–16. doi: 10.1016/S0140-6736(11)61629-2 PMC3254252 22019144

[9] Giuliano AE Hunt KK Ballman KV Beitsch PD Whitworth PW Blumencranz PW. Axillary dissection vs no axillary dissection in women with invasive breast cancer and sentinel node metastasis: a randomized clinical trial. JAMA. (2011) 305:569–75. doi: 10.1001/jama.2011.90 PMC5389857 21304082

[10] Fisher B Anderson S Bryant J Margolese RG Deutsch M Fisher ER. Twenty-year follow-up of a randomized trial comparing total mastectomy, lumpectomy, and lumpectomy plus irradiation for the treatment of invasive breast cancer. N Engl J Med. (2002) 347:1233–41. doi: 10.1056/NEJMoa022152 12393820

[11] Van de Steene J Soete G Storme G. Adjuvant radiotherapy for breast cancer significantly improves overall survival: the missing link. Radiotherapy Oncol. (2000) 55:263–72. doi: 10.1016/S0167-8140(00)00204-8 10869741

[12] Fattahi S Ahmed SK Park SS Petersen IA Shumway DA Stish BJ. Reirradiation for locoregional recurrent breast cancer. Adv Radiat Oncol. (2021) 6:100640. doi: 10.1016/j.adro.2020.100640 33506143 PMC7814100

[13] Merino T Tran WT Czarnota GJ. Re-irradiation for locally recurrent refractory breast cancer. Oncotarget. (2015) 6:35051–62. doi: 10.18632/oncotarget.v6i33 PMC4741508 26459388

[14] Marta GN Hijal T de Andrade Carvalho H. Reirradiation for locally recurrent breast cancer. Breast. (2017) 33:159–65. doi: 10.1016/j.breast.2017.03.008 28395234

[15] Li Q Guan X Wu P Wang X Zhou L Tong Y. Early transmission dynamics in wuhan, China, of novel coronavirus-infected pneumonia. N Engl J Med. (2020) 382:1199–207. doi: 10.1056/NEJMoa2001316 PMC7121484 31995857

[16] W.H. Organization. Coronavirus disease (COVID-19) pandemic . Available online at: https://www.euro.who.int/en/health-topics/health-emergencies/coronavirus-covid-19/novel-coronavirus-2019-ncov.

[17] Gandhi M Yokoe DS Havlir DV. Asymptomatic transmission, the achilles' Heel of current strategies to control covid-19. N Engl J Med. (2020) 382:2158–60. doi: 10.1056/NEJMe2009758 PMC7200054 32329972

[18] Peng SM Yang KC Chan WP Wang YW Lin LJ Yen AM. Impact of the COVID-19 pandemic on a population-based breast cancer screening program. Cancer. (2020) 126:5202–5. doi: 10.1002/cncr.33180 32914864

[19] Spicer J Chamberlain C Papa S. Provision of cancer care during the COVID-19 pandemic. Nat Rev Clin Oncol. (2020) 17:329–31. doi: 10.1038/s41571-020-0370-6 PMC7156894 32296166

[20] Liang W Guan W Chen R Wang W Li J Xu K. Cancer patients in SARS-CoV-2 infection: a nationwide analysis in China. Lancet Oncol. (2020) 21:335–7. doi: 10.1016/S1470-2045(20)30096-6 PMC7159000 32066541

[21] Gradishar WJ Moran MS Abraham J Aft R Agnese D Allison KH. Breast cancer, version 3.2022, NCCN clinical practice guidelines in oncology. J Natl Compr Canc Netw. (2022) 20:691–722. doi: 10.6004/jnccn.2022.0030 35714673

[22] Shah C Al-Hilli Z Vicini F. Advances in breast cancer radiotherapy: implications for current and future practice. JCO Oncol Pract. (2021) 17:697–706. doi: 10.1200/OP.21.00635 34652952

[23] Darby SC Ewertz M McGale P Bennet AM Blom-Goldman U Bronnum D. Risk of ischemic heart disease in women after radiotherapy for breast cancer. N Engl J Med. (2013) 368:987–98. doi: 10.1056/NEJMoa1209825 23484825

[24] Delaney G Jacob S Featherstone C Barton M. The role of radiotherapy in cancer treatment: estimating optimal utilization from a review of evidence-based clinical guidelines. Cancer. (2005) 104:1129–37. doi: 10.1002/cncr.21324 16080176

[25] Helleday T Petermann E Lundin C Hodgson B Sharma RA. DNA repair pathways as targets for cancer therapy. Nat Rev Cancer. (2008) 8:193–204. doi: 10.1038/nrc2342 18256616

[26] Hendry JH Bentzen SM Dale RG Fowler JF Wheldon TE Jones B. A modelled comparison of the effects of using different ways to compensate for missed treatment days in radiotherapy. Clin Oncol (R Coll Radiol). (1996) 8:297–307. doi: 10.1016/S0936-6555(05)80715-0 8934049

[27] Hermens AF Barendsen GW. Changes of cell proliferation characteristics in a rat rhabdomyosarcoma before and after x-irradiation. Eur J Cancer (1965). (1969) 5:173–89. doi: 10.1016/0014-2964(69)90065-6 5770295

[28] Thames HD Jr. Peters LJ Withers HR Fletcher GH. Accelerated fractionation vs hyperfractionation: rationales for several treatments per day. Int J Radiat Oncol Biol Phys. (1983) 9:127–38. doi: 10.1016/0360-3016(83)90089-5 6833014

[29] Zhao F Yang D Li X. Effect of radiotherapy interruption on nasopharyngeal cancer. Front Oncol. (2023) 13:1114652. doi: 10.3389/fonc.2023.1114652 37091186 PMC10116059

[30] Yao JJ Zhang F Gao TS Zhang WJ Lawrence WR Zhu BT. Survival impact of radiotherapy interruption in nasopharyngeal carcinoma in the intensity-modulated radiotherapy era: A big-data intelligence platform-based analysis. Radiother Oncol. (2019) 132:178–87. doi: 10.1016/j.radonc.2018.10.018 PMC12883213 30448002

[31] Bese NS Hendry J Jeremic B. Effects of prolongation of overall treatment time due to unplanned interruptions during radiotherapy of different tumor sites and practical methods for compensation. Int J Radiat Oncol Biol Phys. (2007) 68:654–61. doi: 10.1016/j.ijrobp.2007.03.010 17467926

[32] Bleicher RJ. Timing and delays in breast cancer evaluation and treatment. Ann Surg Oncol. (2018) 25:2829–38. doi: 10.1245/s10434-018-6615-2 PMC6123282 29968031

[33] Laird AK. Dynamics of tumour growth: comparison of growth rates and extrapolation of growth curve to one cell. Br J Cancer. (1965) 19:278–91. doi: 10.1038/bjc.1965.32 PMC2071357 14316202

[34] Pearlman AW. Breast cancer–influence of growth rate on prognosis and treatment evaluation: a study based on mastectomy scar recurrences. Cancer. (1976) 38:1826–33. doi: 10.1002/(ISSN)1097-0142 991096

[35] Tilanus-Linthorst MM Kriege M Boetes C Hop WC Obdeijn IM Oosterwijk JC. Hereditary breast cancer growth rates and its impact on screening policy. Eur J Cancer (Oxford Engl 1990). (2005) 41:1610–7. doi: 10.1016/j.ejca.2005.02.034 15978801

[36] van Rijn J van den Berg J Meijer OW. Proliferation and clonal survival of human lung cancer cells treated with fractionated irradiation in combination with paclitaxel. Int J Radiat oncology biology Phys. (1995) 33:635–9. doi: 10.1016/0360-3016(95)00216-L 7558953

[37] Golden EB Formenti SC Schiff PB. Taxanes as radiosensitizers. Anticancer Drugs. (2014) 25:502–11. doi: 10.1097/CAD.0000000000000055 24335716

[38] Cui L Her S Dunne M Borst GR De Souza R Bristow RG. Significant radiation enhancement effects by gold nanoparticles in combination with cisplatin in triple negative breast cancer cells and tumor xenografts. Radiat Res. (2017) 187:147–60. doi: 10.1667/RR14578.1 28085639

[39] Sherry AD Mayer IA Ayala-Peacock DN Abramson VG Rexer BN Chakravarthy AB. Combining adjuvant radiotherapy with capecitabine in chemotherapy-resistant breast cancer: feasibility, safety, and toxicity. Clin Breast Cancer. (2020) 20:344–352.e1. doi: 10.1016/j.clbc.2020.02.010 32234364

[40] Rutqvist LE. Adjuvant endocrine therapy. Best Pract Res Clin Endocrinol Metab. (2004) 18:81–95. doi: 10.1016/S1521-690X(03)00046-0 14687599

[41] Zeng ZJ Li JH Zhang YJ Zhao ST. Optimal combination of radiotherapy and endocrine drugs in breast cancer treatment. Cancer Radiother. (2013) 17:208–14. doi: 10.1016/j.canrad.2013.01.014 23664221

[42] Kantorowitz DA Thompson HJ Furmanski P. Effect of conjoint administration of tamoxifen and high-dose radiation on the development of mammary carcinoma. Int J Radiat oncology biology Phys. (1993) 26:89–94. doi: 10.1016/0360-3016(93)90177-W 8482635

[43] Wazer DE Tercilla OF Lin PS Schmidt-Ullrich R. Modulation in the radiosensitivity of MCF-7 human breast carcinoma cells by 17B-estradiol and tamoxifen. Br J Radiol. (1989) 62:1079–83. doi: 10.1259/0007-1285-62-744-1079 2605455

[44] Wang J Yang Q Haffty BG Li X Moran MS. Fulvestrant radiosensitizes human estrogen receptor-positive breast cancer cells. Biochem Biophys Res Commun. (2013) 431:146–51. doi: 10.1016/j.bbrc.2013.01.006 23313506

[45] Azria D Belkacemi Y Romieu G Gourgou S Gutowski M Zaman K. Concurrent or sequential adjuvant letrozole and radiotherapy after conservative surgery for early-stage breast cancer (CO-HO-RT): a phase 2 randomised trial. Lancet Oncol. (2010) 11:258–65. doi: 10.1016/S1470-2045(10)70013-9 20138810

[46] Ishitobi M Shiba M Nakayama T Motomura K Koyama H Nishiyama K. Treatment sequence of aromatase inhibitors and radiotherapy and long-term outcomes of breast cancer patients. Anticancer Res. (2014) 34:4311–4. 25075064

